# Comparative Analysis of Classification of Neonatal Bilirubin by Using Various Machine Learning Approaches

**DOI:** 10.7759/cureus.62019

**Published:** 2024-06-09

**Authors:** Priti V Bhagat, Mukesh M Raghuwanshi, Ashutosh D Bagde

**Affiliations:** 1 Computer Engineering, St. Vincent Pallotti College of Engineering and Technology, Nagpur, IND; 2 Computer Technology, Yeshwantrao Chavan College of Engineering, Nagpur, IND; 3 Computer Science and Engineering, S. B. Jain Institute of Technology, Management and Research, Nagpur, IND; 4 Biomedical Engineering, Faculty of Engineering and Technology, Datta Meghe Institute of Higher Education and Research, Wardha, IND; 5 Bio Innovation Lab, Jawaharlal Nehru Medical College, Datta Meghe Institute of Higher Education and Research, Wardha, IND

**Keywords:** tsb, hyper-bilirubin, bilirubin, jaundice, neonatal

## Abstract

Background

Neonatal jaundice poses significant risks to newborn health, necessitating early detection and management. Machine learning (ML) offers promising avenues for improving classification and monitoring, potentially revolutionizing neonatal care.

Materials and methods

A comparative analysis was conducted using various ML algorithms to classify neonatal bilirubin levels. Data were collected from neonatal images, and algorithms were trained and tested using standard methodologies. Performance metrics, including accuracy, precision, and recall, were evaluated to assess algorithm effectiveness.

Results

The Nu-Support Vector Classification (NuSVC) model emerged as the most effective, achieving a testing accuracy of 62.50%, with precision and recall rates of 61.90% and 56.52%, respectively. While variability existed among algorithms, these results highlight NuSVC's potential for clinical application in neonatal jaundice screening.

Conclusion

ML holds promise for improving neonatal jaundice detection and management. The findings suggest that the NuSVC algorithm can enhance screening accuracy, potentially mitigating risks associated with untreated neonatal jaundice. Future research should focus on refining models for broader clinical applicability and integrating ML into decision support systems to improve neonatal care globally.

## Introduction

Neonatal jaundice is a traditional disease that affects the newborn in the first three to five days of life. It causes the disintegration of red blood corpuscles (RBCs) to produce bilirubin as a by-product. It passes through the liver and is excreted by the body in bile or urine. Almost 60% of term neonates and 80% of preterm neonates develop symptoms of jaundice [1]. It is observed that when the bilirubin level exceeds 5 mg/dL, it leads to yellow discoloration of the sclera and skin, and an increase in the level may lead to Kernicterus or cause death [2]. The most common treatment for this increased bilirubin, or hyperbilirubinemia, is either phototherapy or exchange transfusion [3,4]. However the treatment protocol for jaundice is well-defined, but the identification or screening of jaundice condition is always a challenge and requires a clinical expert. The most common technique for the diagnosis is to go with the total serum bilirubin (TSB) in which the blood samples are drawn to measure bilirubin in the serum. Although this technique is considered the gold standard in clinical scenarios, it is time-consuming and may cause treatment delays, resulting in the intricacy of clinical studies.

The classification of neonatal bilirubin levels using machine learning (ML) techniques represents a frontier in artificial intelligence and neonatal healthcare. Neonatal jaundice, characterized by elevated bilirubin levels, is a common condition affecting newborns worldwide. Prompt and accurate detection is crucial to prevent severe neurological damage or other long-term effects. Recent advancements in ML provide promising tools for enhancing the precision and efficiency of bilirubin level classification, which could lead to early detection of affected neonates. However, there is a noticeable gap in the literature concerning the comprehensive comparison and analysis of various ML approaches for classifying neonatal bilirubin levels. Most studies are limited in scope, focusing on singular aspects of bilirubin measurement or employing a narrow range of ML techniques without a broader comparison across available methods.

This research aims to bridge this gap by systematically analyzing multiple machine-learning techniques for classifying neonatal bilirubin levels. By evaluating a range of algorithms on a diverse dataset, this study seeks to identify the most effective ML approaches for predicting neonatal jaundice, thereby offering insights that could inform clinical practices and the development of diagnostic tools. In summary, this work presents a comprehensive evaluation of machine learning algorithms for the accurate classification of neonatal bilirubin levels. This work contributes to advancing neonatal care by integrating cutting-edge artificial intelligence technologies.

Literature review

Hardalaç et al. developed a multivariable linear regression model to classify neonatal jaundice, examining 196 subjects. This included 61 subjects with severe jaundice, where bilirubin levels ranged from 0 to 9.9 mg/dL, and 95 with mild jaundice, with levels between 10 to 30 mg/dL. The subjects' image capture process employed a color card featuring eight color regions. For the model, 30 points were selected from each image and distributed across the card's head, arms, feet, sternum, and color region. This approach led to a classification success rate of 92.5% for their two-class classifier [5]. Similarly, Topaloglu and Sur developed a decision support system for jaundice diagnosis, utilizing a dataset from 300 infants with elevated bilirubin levels and applying data mining algorithms to create decision tree models using C5.0 and J48 algorithms. Twenty-one features were used for decision trees aimed to cover 16 types of illnesses [6].

Polley et al. introduced a method using diffused reflection measurements from the conjunctiva to diagnose jaundice independent of race, age, and gender, utilizing an optical fiber setup capable of measuring light absorption from 400 to 800 nm [7]. Subramanian et al. proposed that light reflections from the skin surface could estimate bilirubin levels [8]. Arulmozhi and Ezhilarasi employed images of newborns to detect jaundice, using a hybrid median filter for image preprocessing and extracting texture and color features through the Gray-Level Co-Occurrence Matrix. Feature redundancy was reduced with the help of the Maximal Information Compression Index [9].

De Greef et al. designed a smartphone application, BiliCam, for jaundice monitoring in neonates, capturing images with a color calibration card during the first hours of life and follow-up periods. The application was trained on five machine learning algorithms using 21 features, achieving a rank order correlation of 0.85 with TSB with a mean error of 2.0 mg/dL [10]. Aydın et al. also developed a non-invasive system using a standard smartphone to capture images for early jaundice diagnosis, achieving an 85% success rate with k-nearest neighbor (kNN) and support vector regression (SVR) machine learning regression on the processed images [5]. Mansor et al. utilized image features like mean, standard deviation, skewness, kurtosis, energy, and entropy to detect neonatal jaundice using a k-NN classifier [11].

Dissaneevate et al. introduced a Mobile Computer-Aided Diagnosis (mCADx) platform, employing decision trees, k-nearest neighbor, and convolutional neural networks (CNNs), with the CNN achieving the highest accuracy [2]. Sammir et al. created a portable monitoring system capturing sclera images with a custom goggle, achieving a 90% accuracy rate [12]. Outlaw et al. developed the neoSCB app to diagnose neonatal jaundice from sclera images, achieving high sensitivity and specificity at specific bilirubin thresholds [13]. Dharmar et al. designed an expert system for jaundice diagnosis by paramedical staff using a rule-based decision tree, requiring constant updates from developers [14]. Padidar et al. designed a smartphone app for estimating bilirubin levels in neonates with varying degrees of sensitivity and specificity, achieving a correlation of 0.479 between estimated and total serum bilirubin levels [15].

Despite extensive efforts towards non-invasive bilirubin detection, achieving definitive outcomes remains challenging. This article aims to contribute to this field by elaborating a comparative analysis of bilirubin classification using various machine learning algorithms such as NuSVC and Random Forest Classifier, detailed in the succeeding sections.

## Materials and methods

This study, comprising 192 neonates affected by bilirubin, was collected from Acharya Vinoba Bhave Rural Hospital in Wardha. Before data collection, approval was secured from the institutional ethical committee. Data collection involved capturing images of all neonates within a controlled environmental setting using two distinct imaging systems: a Google Pixel camera and a Basler Medical grade camera equipped with a Kowa LM12HC F1.4 f12.5mm lens.

The imaging process utilized proprietary software supplied by the camera manufacturers, and each image capture incorporated a manually designed color calibration card. This color calibration card, containing 12 colors such as Dark Skin (115, 82, 68), Light Skin (194, 150, 130), and Blue Sky (98, 122, 157), among others, was strategically positioned below the neonate's sternum while capturing an image. For the Google Pixel camera, a custom application was developed to facilitate the recording of study ID, birth time, and image. The application guides the user to properly place the color calibration card on the neonate's sternum, ensuring the card and the neonate's sternum and forehead are visible in the frame. A "viewfinder" feature helps to align the color calibration card within a predefined area on the screen, prompting the user to take a series of images of neonates.

**Figure 1:**
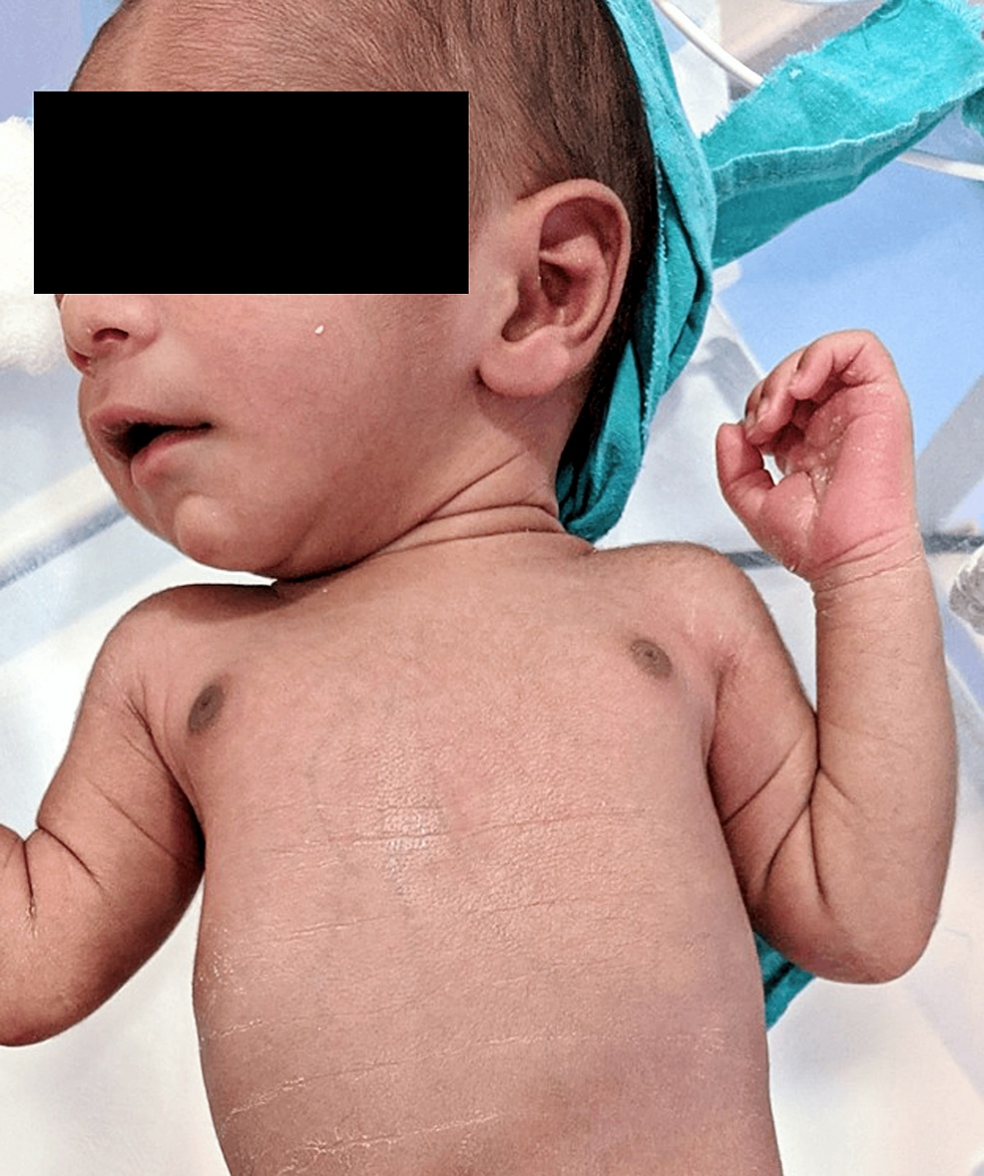
Sample Input Image

Image quality was rigorously assessed by applying a threshold to the standard deviation of pixel values for each color patch on the color calibration card. This process aimed to identify any issues with positioning, obstructions, or inconsistent lighting conditions. If any problem is detected, the application recommends recapturing the image to ensure optimal quality for analysis. Figure 1 shows the sample image collected from the NICU.

Method

The primary goal of this research is to develop a decision support system aimed at early detection of jaundice. This condition is characterized by the yellowing of the skin, a symptom resulting from elevated bilirubin levels. To enhance the detection of skin discoloration, this study involved converting the original RGB (Red, Green, Blue) color values into YCbCr and Lab color spaces. The YCbCr space divides color images into luminance (Y), which represents the brightness levels, and chrominance components (Cb and Cr), indicating color differences. The Lab color space includes L* for lightness and a* and b* for the green-red and blue-yellow color components, respectively.

The conversion equations for YCbCr are as follows:

Y = 0.257R + 0.504G + 0.098B + 16 (1)

Cb = -0.148R - 0.291G + 0.439B + 128 (2)

Cr = 0.439R - 0.368G - 0.071B + 128 (3)

For the Lab components, the equations are:

L = Y1 = 0.2126 * R + 0.7152 * G + 0.0722 * B (4)

A = 1.4749 * (0.2213 * R - 0.3390 * G + 0.1177 * B) + 128 (5)

b = 0.6245 * (0.1949 * R + 0.6057 * G - 0.8006 * B) + 128 (6)

**Figure 2:**
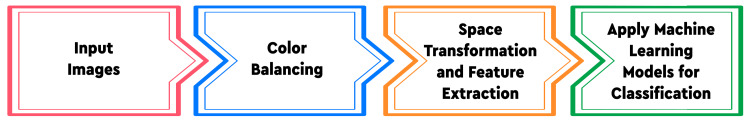
Flowchart for the proposed methodology

Twelve features were considered for comparative analysis, including the means of the RGB channels, YCbCr, and Lab color spaces, and three features derived from the linear gradient of the RGB plane. These features and total serum bilirubin (TSB) values were used to train the ML model. Figure 2 shows the work of the proposed methodology.

The implementation utilized the sci-kit-learn library in Python, facilitating a comprehensive comparison of various classification models. The dataset was divided into training and testing sets for model evaluation, with performance metrics such as Accuracy, Precision, Recall, and Area Under the Curve (AUC) being utilized for assessment. Accuracy reflects the proportion of correctly identified jaundice cases among all the newborns tested, Precision measures the proportion of true positive jaundice detections out of all positive predictions, and Recall indicates the fraction of true positive detections out of all actual jaundice cases.

## Results

The comparative analysis of machine learning algorithms in classifying neonatal bilirubin levels reveals a range of training and testing accuracies, precision, recall, and AUC values. Table 1 shows the overall results of various machine-learning algorithms. Notably, the NuSVC algorithm demonstrated a testing accuracy of 62.50%, with a precision of 61.90% and recall of 56.52%, showcasing a balanced performance in model prediction. The Random Forest Classifier achieves a perfect training accuracy but declines performance in testing to 58.33%, suggesting a potential overfitting during the training phase. On the other hand, algorithms such as the AdaBoost Classifier and ExtraTreesClassifier show more consistency between training and testing accuracies.

**Table 1:** Overall Classification Result MLA: Machine Learning Algorithm; NuSVC: Nu-Support Vector Classification; RandomForestClassifier: Random Forest Classifier; ExtraTreesClassifier: Extra Trees Classifier; AdaBoostClassifier: Adaptive Boosting Classifier; SGDClassifier: Stochastic Gradient Descent Classifier; GaussianProcessClassifier: Gaussian Process Classifier; Perceptron: Perceptron; GaussianNB: Gaussian Naive Bayes; KNeighborsClassifier: k-Nearest Neighbors Classifier; GradientBoostingClassifier: Gradient Boosting Classifier; LogisticRegressionCV: Logistic Regression with Cross-Validation; PassiveAggressiveClassifier: Passive Aggressive Classifier; BaggingClassifier: Bagging Classifier; BernoulliNB: Bernoulli Naive Bayes; LinearSVC: Linear Support Vector Classification; DecisionTreeClassifier: Decision Tree Classifier; RidgeClassifierCV: Ridge Classifier with Cross-Validation; SVC: Support Vector Classifier.

MLA Name	MLA Train Accuracy	MLA Test Accuracy	MLA Precision	MLA Recall	MLA AUC
NuSVC	0.6458	0.6250	0.619048	0.565217	0.622608
RandomForestClassifier	1.0000	0.5833	0.571429	0.521739	0.580870
ExtraTreesClassifier	1.0000	0.5833	0.565217	0.565217	0.582609
AdaBoostClassifier	0.9444	0.5625	0.538462	0.608696	0.584348
SGDClassifier	0.5278	0.5625	0.562500	0.391304	0.555652
GaussianProcessClassifier	1.0000	0.5208	0.500000	0.478261	0.519130
Perceptron	0.4931	0.5208	0.000000	0.000000	0.500000
GaussianNB	0.6111	0.5208	0.500000	0.565217	0.522609
KNeighborsClassifier	0.7222	0.5208	0.500000	0.434783	0.517391
GradientBoostingClassifier	1.0000	0.5000	0.476190	0.434783	0.497391
LogisticRegressionCV	0.5069	0.4792	0.454545	0.434783	0.477391
PassiveAggressiveClassifier	0.5069	0.4792	0.479167	1.000000	0.500000
BaggingClassifier	0.9792	0.4792	0.454545	0.434783	0.477391
BernoulliNB	0.5069	0.4792	0.479167	1.000000	0.500000
LinearSVC	0.5069	0.4792	0.479167	1.000000	0.500000
DecissionTreeClassifier	1.0000	0.4792	0.458333	0.478261	0.479130
RidgeClassifierCV	0.5972	0.4583	0.428571	0.391304	0.455652
SVC	0.5417	0.4375	0.444444	0.695652	0.447826

**Figure 3:**
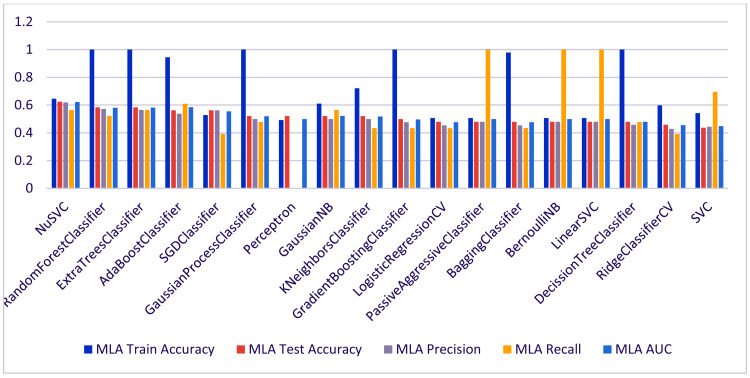
Comparative Analysis of Machine Learning Algorithms

Figure 3 showcases the comparative analysis of various machine-learning algorithms that classify neonatal bilirubin levels. The graph provides a visual comparison, clarifying which algorithms performed better in testing accuracy on the dataset. It is concluded from the bar plot that some algorithms, like NuSVC and Random Forest Classifier, show higher testing accuracy, while other algorithms have scope for improvement. This visual representation supports quickly identifying which models might be more promising for further tuning and application in clinical settings.

## Discussion

The study's findings align with existing literature, suggesting that combining machine learning algorithms and pre-processing methods can significantly enhance the detection and classification of neonatal jaundice. Polley et al. (2015) support the premise that non-contact optical devices coupled with analytical algorithms can effectively monitor jaundice, which corresponds to the higher success rates observed in certain algorithms within this study [7]. In the realm of non-invasive bilirubin measurement, Subramanian et al. (2018) indicate that smartphones can be powerful tools, likely due to their sophisticated sensors and computational capabilities, a concept mirrored in the application of sophisticated machine learning algorithms like NuSVC, which showed strong performance metrics in our analysis [8].

The Random Forest Classifier's training accuracy was exceptional; however, the drop in testing accuracy is indicative of a phenomenon commonly referred to as overfitting. This is where a model performs exceptionally well on the training data but fails to generalize to unseen data, a challenge also discussed in machine learning-based approaches to jaundice detection by Mansor et al. (2013) [16]. Their work emphasizes the need for robust models that perform consistently across different datasets, a criterion that Random Forest did not meet in this instance. Moreover, the precision and recall metrics across models provide insights into the reliability of these classifiers in a clinical context. High precision, as seen in the Passive Aggressive Classifier and the Bagging Classifier, indicates a low false-positive rate, which is crucial in medical diagnoses to avoid unnecessary treatment. In contrast, high recall values suggest that a model is capable of identifying most true positive cases, an attribute that would be vital for a condition where early detection can significantly alter the course of treatment and prognosis.

In summary, the results of this study support a careful consideration of machine learning models' generalizability and predictive performance. These factors are paramount when developing decision support systems for medical applications, such as the early prediction of neonatal jaundice, where the stakes are high and the margin for error is minimal. Further research should focus on refining the performance of these algorithms to enhance their real-world applicability, taking into account the unique challenges presented by neonatal care.

## Conclusions

This study extensively compared machine learning algorithms for classifying neonatal bilirubin levels. NuSVC showed the most balanced performance with 62.50% testing accuracy, suggesting potential for clinical use. Random Forest overfit, while AdaBoost and ExtraTrees showed consistency. Despite promising results, overfitting and real-world testing are concerns. Future research could explore ensemble methods and mobile/cloud applications for wider accessibility, aiming to improve neonatal health globally.
